# Intraoperative nerve monitoring in thyroid and parathyroid surgery: a decade of Italian practice

**DOI:** 10.1007/s13304-025-02157-6

**Published:** 2025-04-01

**Authors:** R. Melcarne, G. Docimo, P. S. L. Aiello, S. Andreani, N. Avenia, G. Basili, C. Bellotti, D. Bettini, M. Biffoni, M. Bononi, A. Bove, P. G. Calò, A. Casaril, G. Cavallaro, M. G. Chiofalo, F. Consorti, C. De Crea, L. De Pasquale, P. Del Rio, C. Dobrinja, L. Giacomelli, G. Graceffa, A. Gurrado, M. Iacobone, N. Innaro, E. Leopaldi, G. Lupone, G. Materazzi, M. Minuto, B. Mullineris, N. Palestini, R. Panconesi, I. Pauna, A. Pezzolla, I. P. Pisano, P. Princi, F. Quaglino, M. Raffaelli, L. Rosato, P. V. Sartori, G. Scerrino, F. Scolari, M. Testini, E. Traini, SIUEC IONM Collaborative Group, G. L. Ansaldo, A. Antonino, S. Beretta, C. Bergamo, E. Bonadies, A. Borasi, A. Borrelli, M. Bossotti, E. Brugger, B. Calì, A. Caracciolo, P. Carcoforo, C. Casella, D. Cavaniglia, D. Chiari, F. D’Angelo, A. De Carlo, G. M. De Luca, G. Di Filippo, G. Di Meo, W. Di Natale, M. G. Esposito, F. Feroci, A. Galimberti, P. Gallucci, A. Garbellini, V. Gatti, L. Giangreco, R. Granata, P. Guarino, E. Iannuzzi, F. Medas, R. Morandi, M. Niederkofler, L. Oragano, N. Osman, N. C. Paladino, F. Palma, P. Papini, A. Pasculli, F. Pedicini, L. Rossi, L. Sessa, A. Tudisco, S. Vanella, E. Varaldo, M. Veroux, T. Zurleni, M. Boniardi

**Affiliations:** 1 Department of Translational and Precision Medicine, Sapienza University of Rome, Viale del Policlinico, 155, 00161 Rome, Italy; 2 Division of Thyroid Surgery, University of Campania “L. Vanvitelli”, Naples, Italy; 3 Endocrine Surgery Unit, Department of General Oncology and Mini-Invasive Surgery, ASST Grande Ospedale Metropolitano Niguarda, 20162 Milan, Italy; 4 General and Endocrine Surgery Unit, S. Maria University Hospital, University of Perugia, 05100 Terni, Italy; 5 General Surgery Department, Endocrine Surgery Unit, Azienda USL Toscana Nord-Ovest, Pontedera, Italy; 6 Dipartimento di Scienze Medico-Chirurgiche e Medicina Traslazionale, Università Sapienza di Roma, Rome, Italy; 7 Endocrine Surgery Unit, “Morgagni-Pierantoni” Hospital, Forli, Italy; 8 UOSD Endocrine Surgery - Department of General and Speciality Surgery, Sapienza University, 00185 Rome, Italy; 9 Department of Surgery, Sapienza University, Rome, Italy; 10 Department of Medicine, Dentistry and Biotechnology, University “G. D’Annunzio”, Chieti, Italy; 11 Department of Surgical Sciences, University of Cagliari, Cagliari, Italy; 12 Endocrine Surgery Unit, Pederzoli Hospital, Peschiera del Garda, Verona Italy; 13 Pathology Unit, Istituto Nazionale Tumori-IRCCS-Fondazione G. Pascale, Naples, Italy; 14 UOC Chirurgia Endocrina, Ospedale Isola Tiberina, Gemelli Isola, Rome, Italy; 15 Centro di Ricerca in Chirurgia delle Ghiandole Endocrine e dell’Obesità, Università Cattolica del Sacro Cuore, Rome, Italy; 16 Thyroid Unit, Department of Health Sciences, Santi Paolo e Carlo Hospital, Università degli Studi di Milano, Milan, Italy; 17 Unit of General Surgery, Department of Medicine and Surgery, University of Parma, Parma, Italy; 18 General Surgery Unit, Department of Medicine, Surgery and Health Sciences, University of Trieste, Cattinara University Hospital, Strada di Fiume 447, 34149 Trieste, Italy; 19 Department of Surgical Oncological and Oral Sciences, University of Palermo, 90127 Palermo, Italy; 20 Department of Precision and Regenerative Medicine and Ionian Area (DiMePre-J), University Medical School of Bari, 70124 Bari, Italy; 21 Endocrine Surgery Unit, Department of Surgery, Oncology and Gastroenterology, Padova University Hospital, Padua, Italy; 22 Unit of Endocrine Surgery, AOU “Dulbecco”, University “Magna Graecia” of Catanzaro, 88100 Catanzaro, Italy; 23 Surgery Department, IGEA Private Hospital, Milan, Italy; 24 ”A. Cardarelli” Hospital, Naples, Italy; 25 Endocrine Surgery Unit, University Hospital of Pisa, Pisa, Italy; 26 Department of Surgical Sciences and Integrated Diagnostics, University of Genoa, IRCCS Ospedale Policlinico San Martino, Genoa, Italy; 27 Unit of General Surgery, Emergency and New Technologies, Modena Hospital, 41126 Modena, Italy; 28 Humanitas - Clinica Fornaca di Sessant Private Hospital, Turin, Italy; 29 Endocrinology Unit, Careggi Hospital and University of Florence, Florence, Italy; 30 Istituto di Patologia Chirurgica AOU, Sassari, Italy; 31 UOC Centro Multifunzionale di Chirurgia Endocrina, Ospedale Cristo Re, Rome, Italy; 32 Department of General Surgery, Maria Vittoria Hospital, ASL City of Turin, Turin, Italy; 33 UOC Chirurgia Endocrina e Metabolica, Fondazione Policlinico Universitario Agostino Gemelli IRCCS, Rome, Italy; 34 Department of Surgery-ASL TO4, Ivrea Hospital, 10015 Ivrea, Italy; 35 General Surgical Department, ASST Brianza-Pio XI Hospital, 20832 Desio, Italy; 36 Endocrine Surgery Unity, Ospedale San Carlo di Nancy GVM, Via Aurelia, 275, 00165 Rome, Italy

**Keywords:** Recurrent laryngeal nerve, Thyroidectomy, Parathyroid surgery, Surgical safety, Nerve protection, Clinical outcomes

## Abstract

Intraoperative nerve monitoring (IONM) has been recognized as a useful adjunct into the field of thyroid and parathyroid surgery, aiding in the accurate identification and preservation of the recurrent laryngeal nerve (RLN), consequently reducing the probability of nerve injury and promoting surgical safety. This investigation seeks to amplify the findings of a similar nationwide survey performed in 2014 by offering an updated assessment of IONM practices among Italian surgical institutions, reflecting a decade of advancements and transformations in clinical approaches. A comprehensive nationwide survey was implemented targeting Italian endocrine surgeons to assess the prevalence, utilization patterns, attitudes and perceptions surrounding IONM in the context of thyroid and parathyroid surgical procedures. Structured questionnaires were administered to seventy endocrine surgery centers, and the resulting data were evaluated employing both quantitative and qualitative analysis methods. The survey disclosed that 67.14% of the participants confirmed the routine deployment of IONM across all cervical surgical procedures, particularly in high-risk contexts. Relative to the findings from 2014, a marked rise in the implementation of IONM has been recorded; however, variability persists, especially between continuous and intermittent monitoring strategies. Surgeons recognized improved surgical safety, decreased complication frequencies, and educational advantages as significant key drivers for the incorporation of IONM. Nonetheless, issues, such as false-positive and false-negative results, along with the lack of standardized protocols, remain barriers to its uniform application. During the past ten years, IONM has been thoroughly adopted by Italian endocrine surgeons as a result of its critical contribution to the improvement of surgical outcomes and the provision of educational resources. However, the results highlight the imperative for further standardization of protocols, the advancement of training programs, and the resolution of reimbursement obstacles to ensure equitable and consistent application of IONM across Italian centers, ultimately optimizing patient care.

**Supplementary Information:**

The online version contains supplementary material available at 10.1007/s13304-025-02157-6.

## Introduction

Intraoperative nerve monitoring (IONM) has been recognized as a crucial element across various surgical specialties, particularly within the domains of thyroid and parathyroid surgical interventions, where it serves a key function in safeguarding the recurrent laryngeal nerve (RLN) and other significant neural structures. The adoption of IONM in these surgical contexts substantially mitigates the risk of RLN impairment, which in turn decreases the incidence of vocal cord paralysis and the complications that follow. This technological innovation not only augments surgical precision and patient safety but also significantly improves functional outcomes and overall quality of life [1].

Historically, the predominant strategy for preventing laryngeal nerve damage during thyroidectomy has involved the visual identification of the RLN and the external branch of the superior laryngeal nerve (EBSLN) throughout the surgical process. Although this technique verifies their anatomical preservation, it fails to evaluate their functional efficacy [2]. The advent of neuromonitoring, which facilitates both anatomical confirmation and functional assessment of the laryngeal nerves, marked a noteworthy progression in the field of thyroid surgery [2, 3].

In the past thirty years, IONM has considerably enhanced the safety and precision of thyroid and parathyroid surgical procedures. IONM facilitates the implementation of staged thyroidectomy in instances where unilateral nerve injury has been detected, thus preventing the manifestation of bilateral vocal fold paresis. The clinical, educational, and legal advantages associated with IONM have driven its widespread utilization on a global scale among both experienced surgeons and surgical residents [4–6].

Despite its well-documented benefits, the implementation and the adoption of IONM exhibit substantial variability across different countries, shaped by factors, such as healthcare systems, surgeon expertise, and regulatory frameworks. In Europe, data derived from the EUROCRINE registry in 2022 (www.eurocrine.eu) indicates that approximately 84% of thyroid surgical procedures are monitored, with 86% employing intermittent neuromonitoring (i-IONM) [7]. In Italy, the IONM landscape reflects a distinctive scenario molded by the country’s healthcare policies, educational programs, and technological innovations [8, 9].

The objective of this study was to explore the contemporary status of IONM utilization in thyroid and parathyroid surgical procedures throughout Italy. The research seeks to determine the frequency of IONM implementation, investigates the endocrine procedures most frequently assisted by IONM, and assesses the perceived merits and difficulties experienced by endocrine surgeons in Italy. Through a comprehensive survey and an analysis of existing data, this research aims to elucidate trends, challenges, and potential strategies for enhancing the integration of IONM within the framework of Italian endocrine surgery.

Gaining insights into the specific dynamics of IONM adoption for thyroid and parathyroid surgeries in Italy not only enriches the broader domain of endocrine surgery but also imparts invaluable knowledge for healthcare practitioners and policymakers. This knowledge is crucial for augmenting surgical safety and effectiveness, ultimately enhancing patient care within the Italian healthcare system.

## Methods

This investigation is based on a  survey exploring the use  of intraoperative neural monitoring﻿ of RLN during thyroid and parathyroid surgery in Italy﻿ . Its build upon a comparable survey conducted in 2014 [9], thereby enhancing its breadth by integrating additional medical institutes and broadening the topics of inquiry. The primary objective was to evaluate changes in the adoption of IONM methodologies, the determinants influencing its application, and the resultant clinical outcomes.

The survey was meticulously designed as a systematic questionnaire consisting of 38 items, which are listed in Appendix 1. It combines closed-ended questions facilitating quantitative frequency analysis alongside open-ended inquiries intended for qualitative thematic exploration. The background information encompassed surgeon demographics, hospital characteristics, specific data related to the volume of thyroid and parathyroid surgeries performed, as well as detailed information regarding the application of IONM techniques, including continuous versus intermittent monitoring and pre- and postoperative laryngeal assessments.

From December 2023 to January 2024, representatives from thirty-five Italian endocrine surgery centers were engaged. Preliminary data were collected through telephonic interviews, and the initial findings were subsequently piloted within the finalized survey. The outcomes of the pilot survey were presented at the "X Workshop di Aggiornamento in Chirurgia Tiroidea e Paratiroidea SIUEC" in Naples on January 19, 2024. Subsequent to this event, a formalized survey, endorsed by the United Italian Society of Endocrine Surgery (SIUEC), was disseminated electronically to the aforementioned centers.

The research was directed at centers spanning northern, central, and southern Italy, encompassing academic, public, and private institutions. Additional centers were identified through the network of the SIUEC, and the participants were predominantly specialist surgeons engaged in general surgery or otolaryngology (ENT) surgery.

The questionnaire was methodically divided into sections addressing the following aspects: demographics and characteristics of the surgical teams (e.g., age, experience); surgical volume (e.g., annual number of thyroidectomies and parathyroidectomies performed); equipment utilized for IONM (e.g., continuous vs. intermittent monitoring); practices surrounding preoperative and postoperative laryngeal examinations; as well as surgeons' perceptions regarding the utility of IONM for both nerve management and medico-legal considerations.

Responses were garnered via a web-based survey platform (Google Forms) and subsequently exported into Microsoft Excel for the purpose of data processing. A mixed-method approach was adopted for data analysis. The quantitative data were evaluated utilizing STATA version 18 software (StataCorp LLC, College Station, TX, 77,845, USA) to compute descriptive statistics, encompassing frequency distributions and cross-tabulations.

For the qualitative data, a thematic analysis was executed on the open-ended responses to elucidate key themes and trends. This analysis aimed to provide in-depth understanding of surgeons' perspectives on IONM and its implications for clinical practice.

## Results

A total of seventy Italian endocrine surgery centers participated in the survey, and their enumeration is provided in Appendix 2. The participants' email addresses were recorded as responses to the initial inquiry. The distribution of the centers was relatively balanced across northern (42.86%), central (30.00%), and southern Italy (27.14%). A predominant proportion of the centers were associated with academic institutions (44.29%), succeeded by hospital environments (34.29%) and private organizations (18.57%) (Table 1).

**Table 1:** Overview of endocrine surgery centers in Italy

Question	Response	Frequency	Percent	Cumulative percent
2. Currently, the center where you practice your surgical activity is located in:	Central Italy	21	30.00	30.00
	Northern Italy	30	42.86	72.86
	Southern Italy or Islands	19	27.14	100.00
	**Total**	**70**	**100.00**	**100.00**
3. The center where you currently work is part of a:	Hospital	24	34.29	34.29
	Private	13	18.57	52.86
	Academic Hospital	31	44.29	97.14
	Other	2	2.86	100.00
	**Total**	**70**	**100.00**	**100.00**
4. The endocrine surgery center where you practice can be defined as:	Other	3	4.29	4.29
	“*Programma di Chirurgia*”	9	12.86	17.14
	“*Unità Operativa Complessa*” (UOC)	34	48.57	65.71
	“*Unità Operativa Semplice*” (UOS)	24	34.29	100.00
	**Total**	**70**	**100.00**	**100.00**
5. Approximately how many years ago was the endocrine surgery center where you work established?	**M (± SE)**	11.64 (± 1.01)	**[95% CI]**	9.62–13.66
6. How many structured surgeons in your center are involved in endocrine surgery?	**M (± SE)**	3.24 (± 1.58)	**[95% CI]**	2.87–3.62
7. What is the average age of the surgeons working in the endocrine surgery center where you practice?	**M (± SE)**	47.14 (± 6.78)	**[95%CI ]**	45.52–48.75
8. In your operating team, is there a preference (which results in more frequent use) for a particular instrument by one or more operators compared to others?	No	38	54.29	54.29
	Not sure	8	11.43	65.71
	Yes	24	34.29	100.00
	**Total**	**70**	**100.00**	**100.00**
9. If you answered "Yes" to the previous question, have you noticed whether this preference for technology is related to age? (e.g., younger age, greater tendency to use technology)	Other	3	12.50	12.50
	No	11	45.83	58.33
	Not sure	6	25.00	83.33
	Yes	4	16.67	100.00
	**Total**	**24**	**100.00**	**100.00**

Overview of the geographical distribution, institutional affiliation, and classification of endocrine surgery centers in Italy, along with data on surgeon characteristics and preferences for instrument use and technology. *M*: Mean; *SE*: Standard Error; *CI*: Confidence Interval.

### Demographic characteristics

Among the respondents representing the seventy Italian endocrine surgery centers, 27% were females. The mean age of surgeons performing endocrine procedures in these centers was 47.14 years (95% CI: 45.52–48.75). Each center employed an average of three endocrine surgeons (95% CI: 2.87–3.62). Endocrine surgery units were predominantly classified as "*Unità Operativa Complessa*" (48.57%) or "*Unità Operativa Semplice*" (34.29%) (Table 1).

### Surgical volume 

A predominant number of medical institutions reported that they perform between 50 and 150 total thyroidectomies annually (35.71%), while a limited portion of institutions indicated that they conduct more than 500 procedures each year (2.86%). Furthermore, a significant 71.43% of the institutions performed fewer than 50 parathyroidectomies in a year (Table 2).

**Table 2:** Endocrine surgery volumes and trends in IONM utilization

Question	Response	Frequency	Percent	Cumulative percent
10. How many total thyroidectomies are approximately performed in your center per year?	≤ 50	7	10.00	10.00
	> 50 ≤ 150	25	35.71	45.71
	> 150 ≤ 250	20	28.57	74.29
	> 250 ≤ 350	10	14.29	88.57
	> 350 ≤ 500	6	8.57	97.14
	> 500	2	2.86	100.00
	**Total**	**70**	**100.00**	**100.00**
11. How many parathyroidectomies are approximately performed in your center per year?	≤ 50	50	71.43	71.43
	> 50 ≤ 150	18	25.71	97.14
	> 150 ≤ 250	1	1.43	98.57
	> 500	1	1.43	100.00
	**Total**	**70**	**100.00**	**100.00**
12. How many neck dissections are approximately performed in your center per year?	≤ 50	60	85.71	85.71
	> 50 ≤ 150	9	12.86	98.57
	> 350 ≤ 500	1	1.43	100.00
	**Total**	**70**	**100.00**	**100.00**
13. How many endocrine surgeries (in general) do you perform with your team per month? Please indicate a number	**M (± SE)**	26.36 (± 38.73)	**[95% CI]**	17.12–35.59
14. If you consider your endocrine surgery activity (and that of your team) over the past 10 years, do you believe that the use of IONM of the recurrent laryngeal nerve is now more widely used in surgical practice compared to 2014?	No	5	7.14	7.14
	Yes	62	88.57	95.71
	About the same	3	4.29	100.00
	**Total**	**70**	**100.00**	**100.00**

Summary of annual surgical volumes for thyroidectomies, parathyroidectomies, and neck dissections, along with monthly endocrine surgeries and the increased use of IONM since 2014. *M*: Mean; *SE*: Standard Error; *CI*: Confidence Interval.

﻿

### Factors Driving the Increased Use of IONM and Current Practices  

 Furthermore, a significant 88.57% of the participants acknowledged that the application of IONM for RLN management has experienced a notable increase in prevalence over the past decade compared to the year 2014 (Table 2).

The qualitative thematic analysis performed on the open-ended responses to Question n. 15 revealed a multifaceted range of themes, which included the perceived improvement of patient safety, enhanced surgical accuracy, concerns regarding a potential decline in surgical skills, necessary training, and its impact on the interaction between surgeons and patients (Table 3).

**Table 3:** Thematic analysis of endocrine surgery practices and IONM use

15. What do you think are the reasons that have made the use of RLN IONM more common in routine endocrine surgery practice? Please list at least three main reasons		
Theme	Subtheme	Examples
Increases safety	For the patient	Reduces morbidity; prevents bilateral nerve injuries; allows functional prognostic evaluation; identifies the cause of nerve LOSS; avoids tracheotomy; allows immediate management of complications; requested by the patient
	For the surgeon	Protects against medico-legal disputes; provides demonstrability through recordings; increases confidence in complex surgeries; offers greater peace of mind in managing postoperative dysphonia; habitual use
Advantages of use	Ease of use and handling	
	Educational	Reduces the learning curve; optimizes and standardizes the procedure
	Improved anatomy definition	In complex cases (recurrences, reoperations, embedded goiters); when there is no anatomical-functional correspondence; in the search for the superior laryngeal nerve (NLS)
	Reduces time	Optimizes and standardizes the procedure
Increases quality	Routine use improves performance	
	Modulates strategy	Indication for two-staged thyroidectomy
	Evolution	Acceptable and decreasing costs; improving technology
	Evidence-based procedure	
External factors	Driven by manufacturers	
	Followed by everyone	
	Accepted by administrations	
	Institutional requirement and adherence to guidelines	
	Used for research purposes	
	Contrary factors	Additional costs, decrease in surgical skills

Summary of key themes from open-ended responses regarding surgical practices and the use of intraoperative neural monitoring (IONM) in endocrine surgery

In the context of preoperative laryngeal examination techniques, 81.43% of the participants indicated the habitual utilization of direct fiberoptic laryngoscopy, while 11.43% applied it in a selective manner, and 5.71% integrated routine indirect fiberoptic laryngoscopy with selective direct laryngoscopy. A mere 1.43% acknowledged the application of routine laryngeal ultrasound in conjunction with selective laryngoscopy (Table 4).

**Table 4:** Preoperative assessment methods and informed consent in thyroid surgery centers

Question	Response	Frequency	Percent	Cumulative percent
16. Which of the following preoperative assessment methods best describes your approach to laryngeal examination before thyroid surgery?	Selected cases of direct fiberoptic laryngoscopy	8	11.43	11.43
	Routine direct fiberoptic laryngoscopy	57	81.43	92.86
	Routine indirect fiberoptic laryngoscopy and, in selected cases, direct fiberoptic laryngoscopy	4	5.71	98.57
	Routine laryngeal ultrasound and selected cases of laryngoscopy*	1	1.43	100.00
	**Total**	**70**	**100.00**	**100.00**
18. In your center, who provides informed consent to the patient before surgery?	Endocrine surgery unit coordinator	23	32.86	32.86
	Resident under the supervision of an attending physician	15	21.43	57.14
	Resident on their own	2	2.86	60.00
	Attending physician	30	42.86	100.00
	**Total**	**70**	**100.00**	**100.00**
19. In your center, do you use the informed consent form provided by the Italian Society of Surgery (SIC)?	No	19	27.14	27.14
	Yes	36	51.43	78.57
	Yes, but with some modifications	15	21.43	100.00
	**Total**	**70**	**100.00**	**100.00**
22. Do you believe that informing the patient about the use of intraoperative nerve monitoring during surgery helps make your communication more effective?	Indifferent	8	11.43	11.43
	No	5	7.14	18.57
	Yes	57	81.43	100.00
	**Total**	**70**	**100.00**	**100.00**
23. Do you believe that informing the patient about the use of intraoperative nerve monitoring during surgery reassures the surgeon?	Indifferent	16	22.86	22.86
	No	14	20.00	42.86
	Yes	40	57.14	100.00
	**Total**	**70**	**100.00**	**100.00**
24. Do you believe that informing the patient about the use of intraoperative nerve monitoring during surgery reassures the patient?	Other (e.g., "maybe")	2	2.86	2.86
	Indifferent	6	8.57	11.43
	No	2	2.86	14.29
	Yes	60	85.71	100.00
	**Total**	**70**	**100.00**	**100.00**

Summary of preoperative laryngeal assessment methods, informed consent practices, and the perceived impact of informing patients about intraoperative nerve monitoring

*The response was recorded as "Other" and corresponded to the open-ended answer given to question 17: “If you selected "Other" as your answer to the previous question, could you briefly provide the response you consider appropriate?”—the only response reported among the 70 responses

The management of informed consent was predominantly conducted by attending physicians (42.86%) and coordinators within the endocrine surgery unit (32.86%). The involvement of residents was comparatively minimal, with 21.43% acquiring consent under supervision and 2.86% doing so autonomously. More than half of the institutions (51.43%) utilized the informed consent document provided by the Italian Society of Surgery (SIC), whereas 21.43% made alterations to it and 27.14% opted not to employ the SIC form (Table 4).

Patient communication supported by IONM was assessed positively, with 81.43% believing that it augmented the effectiveness of communication. Moreover, 57.14% of the respondents reported that the procedure provided reassurance to the surgeon, whereas 85.71% affirmed that it offered reassurance to the patient (Table 4).

Table 5 delineates a thematic analysis of the feedback concerning modifications made to the informed consent document provided by the Italian Society of Surgery (SIC).

**Table 5:** Thematic analysis of modifications to the informed consent form provided by the italian society of surgery (SIC)

20. If you felt the need to make any changes, could you briefly indicate what they are?
Parathyroid reimplantation
Two-stage thyroidectomy
Neuromonitoring
Less frequent complications (tracheal injuries; esophageal injuries)
Possibility of post-surgical residue, including in extracervical locations
Risk percentages for each listed complication
Attach the signed surgical sketch

Table 6 presents a thematic analysis of the responses to Question n. 21, which explores open-ended responses related to methodologies of risk communication.

**Table 6:** Thematic analysis of open-ended responses to risk communication approaches

21. During the informed consent process for surgery, what is your approach to *communicating the risk* of recurrent laryngeal nerve damage? Please briefly describe the key phrases you use in your communication. Kindly list at least three key phrases, in order of importance	
Theme	Examples of expressions and key phrases
How probability is expressed	Possible but not probable; complications below 4%; rarity of the event; frequency of damage; unlikely; general incidence (from literature); personal case incidence; low but not minimal risk; remote risk; minimizable but not eliminable risk
Communicated consequences and corrections	Paresis (with potential for recovery); paralysis (with potential for recovery); tracheostomy; dysphonia; hypophonia and subsequent speech therapy; dysphagia; dyspnea; ICU stay, impact on quality of life; debilitating if bilateral deficit
Advantages	Real-time strategy modification (two-staged thyroidectomy), safety, serves as a guide for the procedure, reduces bilateral nerve injury risk, acceptable surgical risk relative to the appropriateness of the indication, prevention of damage, expert and delicate maneuvers, immediate damage assessment
Key elements and mechanisms	Number of cases treated annually by the center; intraoperative protocol; predisposing conditions that increase risk (including neck dissections and recurrences); nerve function and pathophysiology (including traction damage); experience builds confidence; potential effects of surgical manipulation; nerve position in contact with the thyroid; postoperative hemorrhage increases the risk of RLN damage
Temporal elements	Temporary; definitive; transient; reversible; recovery time
Methods for explanation	Use of anatomical models; hand gestures to simulate laryngeal function; anatomical charts

### Challenges and decision-making

The majority of surgeons indicated that patients rarely inquire about the application of IONM (47.14%) or never pose such questions (21.43%). IONM was utilized in all surgical procedures by 67.14% of the participants, while 14.29% employed it in a selective manner (Table 7).

**Table 7:** Effective use and perceptions of intraoperative neuromonitoring (IONM) in thyroid surgery among surgeons

Question	Freq.	Percent	Cumulative Percent
25. Do patients ever ask if intraoperative neuromonitoring (IONM) will be used for the type of surgery they will undergo?			
No, never	15	21.43%	21.43%
Rarely	33	47.14%	68.57%
Yes, sometimes	16	22.86%	91.43%
Yes, often	6	8.57%	100.00%
Total	70	100.00%	
28. Do you use neuromonitoring of the RLN during all neck endocrine surgeries?			
No, I used it a few times but have stopped	2	2.86%	2.86%
No, I use it selectively	10	14.29%	17.14%
No, never (negative experience)	2	2.86%	20.00%
No, I have not tried it	1	1.43%	21.43%
No, I don’t always use it, but I use it often	8	11.43%	32.86%
Yes, in all operations	47	67.14%	100.00%
Total	70	100.00%	
31. Do you use neuromonitoring during thyroidectomy to identify the RLN?			
No	32	47.76%	47.76%
Yes	35	52.24%	100.00%
Total	67	100.00%	
32. Do you use neuromonitoring during thyroidectomy to “confirm” the RLN (location, integrity, course, etc.)?			
No	2	2.99%	2.99%
Yes	65	97.01%	100.00%
Total	67	100.00%	
33. Do you use neuromonitoring in “continuous” or “intermittent” mode?			
Depends	19	28.36%	28.36%
Continuous only	2	2.99%	31.34%
Intermittent only	46	68.66%	100.00%
Total	67	100.00%	
35. Based on your personal experience and that of your team using IONM of the RLN in thyroid and parathyroid surgery, do you recall cases of false positives (i.e., nerve appears “damaged” but no postoperative paralysis) and/or false negatives (i.e., nerve appears “healthy” but postoperative vocal cord paralysis)?			
No	14	20.90%	20.90%
Don’t remember	2	2.99%	23.88%
Yes	51	76.12%	100.00%
Total	67	100.00%	
36. If you answered “yes” to the previous question, could you indicate the percentage of times this occurred in the past year?	Mean: 3.73	Median: 2.0	Std. dev: 3.78
37. During a total thyroidectomy, if the IONM indicated a recurrent laryngeal nerve (RLN) “lesion” during the lobectomy step on the first lobe, what would be your approach?			
Other	5	7.35%	7.35%
Based on the patient’s clinical situation, I decide with my team whether to continue, even in case of obvious anatomical damage and signal loss	13	19.12%	26.47%
I stop and schedule a “two-stage thyroidectomy”	44	64.71%	91.18%
I carefully evaluate the nerve “visually” and decide with my team whether to continue (e.g., if the nerve appears “intact,” I choose to continue)	6	8.82%	100.00%
Total	68	100.00%	

Italian surgeons use intraoperative neuromonitoring (IONM) in thyroid and parathyroid surgeries

The feedback pertaining to Question n. 26 (‘What do you consider to be the main benefit of laryngeal nerve monitoring?”) and Question n. 27 (which permitted respondents to elaborate on additional advantages not addressed in the prior question) is illustrated in Table 8.

**Table 8:** Responses to Questions n. 26 and n. 27 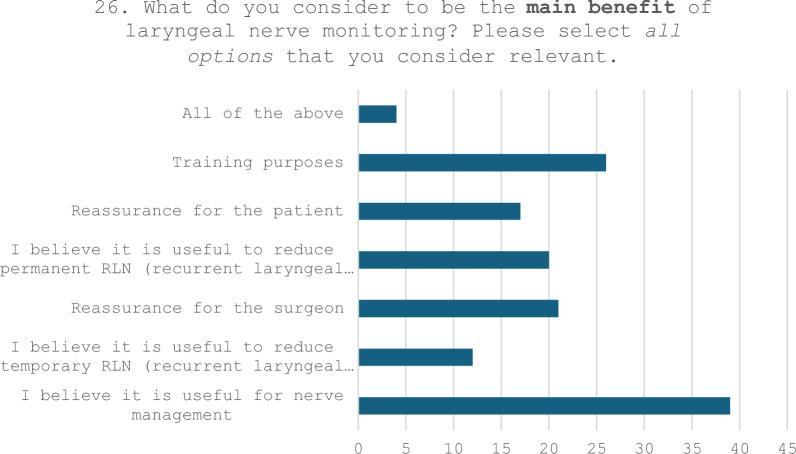

27. Do you believe that a relevant option was not listed among those provided in the previous question? If so, please provide a brief response indicating which option you think should be added to the list
Avoid bilateral paralysis
Quality control
Decision-making for the two-stage thyroidectomy procedure
Legal protection
Certainty of identifying the RLN (at least the motor branch/branches)
Prevent stretch injury (continuous monitoring)

The answers to Question n. 29 (“In which situations do you use RLN IONM?”) and Question n. 30 (“If your use of the device is selective, meaning you do not use the tool in every surgical procedure, in which cases do you consider it essential?”) are presented in Table 9.

**Table 9:** Responses to Questions n. 29 and n. 30 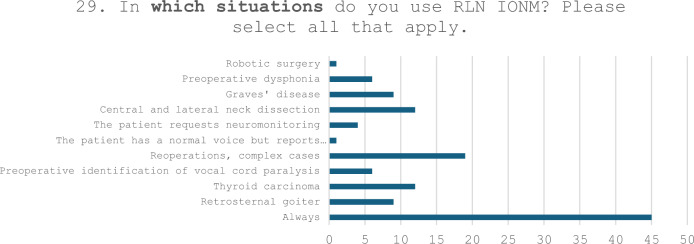

30. If your use of the device is *selective*, meaning you do not use the tool in every surgical procedure, in which cases do you consider it *essential*? Please describe up to *three cases* where you believe the use of RLN IONM is indispensable
Reoperations/recurrences
Suspected neoplastic infiltration of the nerve/preoperative paralysis
Neck dissections
Pediatric patient
Advanced carcinomas/complex cases
Mediastinal goiters

In the context of thyroidectomies, IONM is predominantly employed to validate the integrity of the RLN, with 97.01% utilizing it for this confirmation although only 52.24% incorporate it routinely for RLN identification (Table 7).

Concerning the modalities of IONM (Question n. 33), 68.66% of institutions reported employing intermittent IONM, whereas 28.36% indicated that their selection is contingent upon particular circumstances (Table 10, Question n. 34).

**Table 10:** Responses to Question n. 34

34. If you answered 'it depends,' could you briefly explain in which situations you prefer to use one method over the other? Please describe briefly when you use continuous neuromonitoring versus intermittent, providing your reasoning	
Continuous	In complex oncological cases with potential nerve infiltration
	Recurrent goiter
	Cervico-mediastinal goiter (traction injury)
	Neck dissections
	Previous recurrent nerve injury
	Reoperations
	Lobectomies
Intermittent	Only if the device for continuous monitoring is unavailable
	Complex goiters where access to the vagus nerve is complicated
	Bilateral surgery

False positives or negatives were reported by 76.12% of the respondents, with a median incidence rate of 2% over the past year. 

In instances of total thyroidectomy, a significant proportion of respondents (64.71%) indicated that they would stop the surgical procedure and plan for a two-stage thyroidectomy if IONM revealed nerve injury during lobectomy on the initial side. Conversely, 19.12% reported that they would proceed with the surgery following a meticulous evaluation of the clinical situation and the visual integrity of the nerve (Table 7).

The responses to Question n. 38 are documented in Table 11.

**Table 11:** Responses to Question n. 38

38. Do you think that any relevant statement was not included in the previous question's options? If yes, please provide a brief answer explaining what assessment you think should be added.	
Surgical indication	I stop in cases of benign pathology, and I assess case by case in the presence of advanced carcinoma/severe comorbidities/difficulties in reintubation
Time	I wait, reassess the correct functioning of the NIM (position of the ET tube, electrodes, probe), and if after 10–15 min there is no signal recovery, I stop and schedule a two-stage procedure
Laryngeal twitch	In cases where the nerve is anatomically intact, I wait 40 min. If I have a valid laryngeal twitch (from vagal stimulation), I proceed to the contralateral side with extreme caution and possibly perform a near-total on the contralateral side. If the laryngeal twitch is absent from vagal stimulation with 3–5 A, I stop and schedule a two-stage procedure

## Discussion

This investigation provides critical insights into current practices, trends, and perceptions prevalent among surgeons operating within leading Italian endocrine surgical institutions, with particular emphasis on the the extensive adoption of IONM for the RLN within SIUEC-affiliated units. Over the past decade, a substantial 88.57% of these institutions have incorporated IONM into their surgical protocols (Question n. 14). Presently, IONM serves as a fundamental element of endocrine surgery, being employed in all cervical surgical procedures by 67.14% of these centers (Question n. 28). This phenomenon accentuates the clinical benefits that a majority of Italian surgeons attribute to IONM, notably its contribution to enhancing surgical safety through dependable nerve identification and functional evaluation, particularly in high-risk scenarios [10–12], mitigating postoperative complications, averting bilateral nerve injuries through a staged thyroidectomy strategy [13, 14], and providing educational advantages in the training of junior surgeons [15].

In 2014, prompted by concerns that the prevalence of neuromonitoring during thyroid surgeries may have been underestimated in Italy, the first collaborative Italian survey was conducted to clarify and standardize the protocols for IONM application, management, and documentation in thyroid surgery. Currently, our survey, which covered seventy endocrine surgery centers throughout Italy (42.86% in the northern region, 30% in the central region, and 27.14% in the southern region and the Islands), illustrates that the incidence of RLN monitoring has progressively escalated on a national scale. The prevalence of IONM surged from 1% in 2007 to 10–13% in 2014 [9] and has now experienced exponential growth, attaining 67.14% for neck surgeries. Consequently, whereas in 2013, the majority of Italian surgeons reported minimal use of RLN monitoring, this trend has significantly reversed.

This progression is congruent with international data, wherein the proportion of nerve monitoring application fluctuates between 40 and 90% [16–22]. In 2013, the absence of robust data limited a comprehensive analysis of the factors shaping the adoption of IONM in Italy. At that juncture, this escalation was postulated to arise from a confluence of factors, including the availability of non-invasive IONM devices, enhanced safety, evidence-based clinical benefits, medico-legal implications, educational and research motivations, endorsements from surgical societies, and commercial initiatives [9].

However, the thematic analysis pertaining to Question n.15 elucidates recurring elements that contemporary surgeons now identify as the predominant motivations for the extensive implementation of this technique. The foremost incentive centers around “**improved patient safety**,” which is comprehended as a diminution in morbidity, the prevention of bilateral injuries (thereby circumventing the necessity for tracheotomy), and the expeditious management of complications. Furthermore, IONM provides substantial “**safety benefits for the surgeon**”, encompassing medico-legal protection, augmented confidence in intricate procedures, and heightened familiarity with the device [13, 23].

The concept of “safety” linked to the capability to “modulate” surgical approaches manifests as a constructive theme accentuated throughout various survey responses. For instance, Question n. 37 reveals that 64.71% of surgeons would opt to suspend total thyroidectomy and implement a staged approach should IONM signal RLN injury during the initial lobe, exemplifying the protocol's significant impact on surgical decision-making [24]. These revelations align with contemporary investigations, including the INMSG survey, which emphasize the crucial function of IONM in staged surgical procedures, particularly in scenarios where signal loss is detected on the initial side of a planned bilateral operation [25]. Additionally, research conducted by Ramesh et al. (2024) further demonstrates the effectiveness of IONM in diminishing postoperative vocal cord palsy, particularly in instances of early signal loss, thereby advocating for staged surgical approaches in high-risk patients [26].

In reality, approximately 28% of surgeons continue to base their RLN management protocols on anatomical knowledge, routine visual identification, collaborative clinical evaluation, and experiential factors [27], particularly in contexts where the prevailing inclination is to pause (Question n.37), preserving a robust conviction that “visual nerve identification” continues to be the benchmark for recurrent laryngeal nerve management in thyroid procedures [28].

In the 2014 survey conducted by Dionigi et al. [9], medico-legal concerns emerged as the principal rationale (30%) for surgeons' utilization of IONM [29, 30]. This observation can be ascribed to various clinical considerations, particularly the efficacy of IONM in averting permanent nerve injury, as documented in numerous studies [23–25], mitigating severe patient injuries such as bilateral RLN paralysis [19, 25, 26], and facilitating the recording of normal neurophysiological vagal signals at the conclusion of the surgical intervention. Nonetheless, these aspects are now reframed more positively as “safety” advantages for both patients and surgeons. Despite the propensity for IONM to alleviate the medico-legal liability of the surgeon and diminish economic repercussions for the patient, healthcare system, and insurance entities [31], the significance of RLN IONM within medico-legal contexts continues to be a subject of contention [32–35] and does not constitute the central theme of this survey.

Emerging motivations for the increased adoption of IONM can be succinctly encapsulated as "**practical benefits**": its user-friendliness, enhancement of anatomical visualization, reduction of surgical duration (thereby optimizing and standardizing operative procedures), and provision of educational value, which assists in the learning trajectory for junior surgeons. As early as 2014, Dionigi et al. observed that academic institutions in Italy exhibited a greater propensity to utilize IONM, as these establishments frequently manage complex cases and incorporate IONM into their resident training and educational curricula [9, 15, 36]. Contemporary data reveals a more widespread increase in the utilization of IONM across a diverse array of healthcare institutions, extending beyond the confines of university settings. This broader integration can be attributed to legislative reforms that have augmented training opportunities for junior surgeons in community hospitals, alongside the expanded dissemination of IONM technology itself [37, 38]. Fassari et al. documented that residents engaged with IONM experienced heightened confidence and proficiency in RLN identification, notwithstanding the fact that IONM does not significantly influence safety or complication rates. While IONM does not substantially modify the learning curve or affect outcomes related to safety and complications, it does enhance the confidence of trainees, thereby rendering it an invaluable resource in surgical education [15].

Surgeons also underscore the "**enhanced quality of practice**" as a pivotal element propelling the spread of IONM: its routine implementation enhances performance, facilitates customized strategies (for instance, two-stage thyroidectomy), and promotes "evidence-based" surgical procedures. Nevertheless, survey findings indicate that, despite its assimilation as a normative practice, the necessity for standardized IONM protocols remains evident. For instance, challenges, such as false positives and negatives, reported by 76.12% of respondents, accentuate the imperative for continuous technical refinement and training to bolster diagnostic precision. This requisite for structured training is further corroborated by Wu et al. [31], who stress the significance of formal IONM training programs to enhance accuracy and consistency in clinical practice [5]. Variability in application continues to exist, particularly between continuous and intermittent modalities (Question n.33), highlighting the necessity for the establishment of consistent guidelines across endocrine surgery centers in Italy. Such variability implies an urgent need for further standardization, with prospective initiatives concentrated on the formulation of national guidelines and the expansion of IONM training programs to foster consistency and ameliorate patient outcomes.

Ultimately, the “**external factors**” identified, including “industry influence,” the inclination to “align with others” (“as it is widely utilized”), and the belief that it “diminishes surgical proficiency,” were also acknowledged as contributing factors to the heightened adoption in the preceding decade.

A notable dimension unveiled by the survey pertains to surgeons’ perspectives on **physician–patient communication** concerning the utilization of IONM. According to the Consensus Statement from the International Neural Monitoring Study Group (2021), it is imperative for the surgeon to supply patients with clear and significant information regarding IONM, incorporating its advantages and disadvantages, while accounting for the patient’s educational experience, emotional state, and understanding capacity. Survey results demonstrate that surgeons generally regard informing patients about IONM as a means to enhance the efficacy of communication (81.43%—Question n.22) and to provide reassurance to both themselves (57.14%—Question n.23) and patients (85.71%—Question n.24)—with a significant proportion of patients (31.43%) inquiring about the device (Question n.25). Furthermore, modifications to the informed consent process to incorporate neuromonitoring have been documented (Question n.20). The qualitative responses to Question n.21 elucidated the varied strategies employed by surgeons in discussing the risk to the RLN, utilizing probabilistic terminology, such as “possible but not probable,” “less than 4%,” “rarity of event,” and “risk frequency,” thereby underscoring the difficulty of accurately conveying probabilistic language [39].

Conveying risk represents a multifaceted challenge for surgeons, who must navigate patients’ anxieties and expectations during informed consent discussions pertaining to surgical interventions [40]. This procedure frequently necessitates the translation of probabilistic terms—such as "possible," "may," "could," "uncertain," "likely," "unclear," among others—which are intrinsically ambiguous, into quantifiable values for informed decision-making [41, 42].

Surgeons also indicate that they discuss the potential consequences and remedial actions associated with risks, encompassing “stupor and/or paralysis (with potential for recovery),” “tracheotomy,” “dysphonia,” “hypophonia necessitating speech therapy,” “dysphagia,” “dyspnea,” and the possibility of “transfer to intensive care,” or “impact on quality of life,” while elucidating the “temporal nature” of these effects (e.g., temporary, permanent, transient, reversible, and estimated recovery duration). To facilitate comprehension, they may employ “anatomical models or diagrams,” simulate laryngeal function through manual gestures, articulate the advantages of utilizing the device, and delineate risk-inducing factors (e.g., predisposing elements, such as lymphadenectomy and recurrent disease, nerve positioning, or postoperative hemorrhage).

In conclusion, there persist certain objective concerns regarding the further expansion of IONM utilization in Italy. As noted by Dionigi et al. in 2014, a particular rationale for the current 67.14% adoption rate of RLN IONM potentially not reaching 100% is that the reimbursement structure for procedures in Italy (Diagnosis-Related Group, DRG 290) remains configured in a manner that fails to incentivize the incorporation of novel technologies or methodologies in thyroid surgery. The adoption of IONM is constrained by inadequate reimbursement for thyroid procedures, a deficiency of qualified anesthetists, limited collaborative efforts between anesthetists and surgeons, restricted availability of equipment, and a paucity of evidence derived from randomized studies.

## Conclusion

This investigation elucidates the substantial increase in the application of IONM within the realm of Italian endocrine surgery, thereby reaffirming its crucial contribution to the enhancement of surgical safety and precision, particularly in scenarios characterized by elevated risk. Nevertheless, it is essential to note that no existing system currently assures the absolute prevention of nerve injury. This highlights the indispensable significance of the surgeon's expertise, the resources provided by high-volume surgical centers, and the adoption of rigorous surgical techniques in attaining optimal postoperative outcomes. Moreover, the identified discrepancies in clinical practice accentuate the imperative for increased standardization, the formulation of uniform practice guidelines, and the development of specialized training programs, particularly aimed at junior surgeons in the course of their specialized training. Adressing the challenges associated with procedural reimbursement will also be vital for ensuring equitable quality of care across Italian medical centers and for further enhancing patient outcomes.

## Supplementary Information

Below is the link to the electronic supplementary material.

Supplementary file1 (PDF 326 KB)

Supplementary file2 (DOCX 10 KB)

## Data Availability

Data supporting the findings of this study are available from the corresponding author upon reasonable request.

## References

[1] Malik R, Linos D (2016) Intraoperative neuromonitoring in thyroid surgery: a systematic review. World J Surg 40(8):2051–2058. 10.1007/s00268-016-3594-y 27329143 10.1007/s00268-016-3594-y

[2] Wojtczak B, Sutkowska-Stępień K, Głód M, Kaliszewski K, Sutkowski K, Barczyński M (2024) Current knowledge on the use of neuromonitoring in thyroid surgery. Biomedicines 12(3):675. 10.3390/biomedicines12030675 38540288 10.3390/biomedicines12030675 PMC10968482

[3] Pardal-Refoyo JL, Parente-Arias P, Arroyo-Domingo MM, Maza-Solano JM, Granell-Navarro J, Martínez-Salazar JM, Moreno-Luna R, Vargas-Yglesias E (2018) Recommendations on the use of neuromonitoring in thyroid and parathyroid surgery. Acta Otorrinolaringol Esp (Engl Ed) 69(4):231–242. 10.1016/j.otorri.2017.06.005 28917827 10.1016/j.otorri.2017.06.005

[4] Schneider R, Randolph GW, Dionigi G, Wu CW, Barczynski M, Chiang FY, Al-Quaryshi Z, Angelos P, Brauckhoff K, Cernea CR, Chaplin J, Cheetham J, Davies L, Goretzki PE, Hartl D, Kamani D, Kandil E, Kyriazidis N, Liddy W, Orloff L, Scharpf J, Serpell J, Shin JJ, Sinclair CF, Singer MC, Snyder SK, Tolley NS, Van Slycke S, Volpi E, Witterick I, Wong RJ, Woodson G, Zafereo M, Dralle H (2018) International neural monitoring study group guideline 2018 part I: Staging bilateral thyroid surgery with monitoring loss of signal. Laryngoscope 128(Suppl 3):S1–S17. 10.1002/lary.27359 30289983 10.1002/lary.27359

[5] Wu CW, Dionigi G, Barczynski M, Chiang FY, Dralle H, Schneider R, Al-Quaryshi Z, Angelos P, Brauckhoff K, Brooks JA, Cernea CR, Chaplin J, Chen AY, Davies L, Diercks GR, Duh QY, Fundakowski C, Goretzki PE, Hales NW, Hartl D, Kamani D, Kandil E, Kyriazidis N, Liddy W, Miyauchi A, Orloff L, Rastatter JC, Scharpf J, Serpell J, Shin JJ, Sinclair CF, Stack BC Jr, Tolley NS, Slycke SV, Snyder SK, Urken ML, Volpi E, Witterick I, Wong RJ, Woodson G, Zafereo M, Randolph GW. International neuromonitoring study group guidelines 2018: Part II: Optimal recurrent laryngeal nerve management for invasive thyroid cancer-incorporation of surgical, laryngeal, and neural electrophysiologic data. Laryngoscope. 2018;128 Suppl 3:S18-S27. 10.1002/lary.27360 10.1002/lary.27360 30291765

[6] Zhu Y, Gao DS, Lin J, Wang Y, Yu L (2021) Intraoperative neuromonitoring in thyroid and parathyroid surgery. J Laparoendosc Adv Surg Tech A 31(1):18–23. 10.1089/lap.2020.0293 32614658 10.1089/lap.2020.0293

[7] Testini M, Gurrado A (2024) Thyroid surgery. Updates in Surgey, Springer 31146-8. 10.1007/978-3-031-31146-8

[8] Dionigi G, Calò PG, Materazzi G et al (2020) Neuromonitoraggio intraoperatorio in chirurgia tiroidea: considerazioni nell’ambito della Società Italiana Unitaria Endocrinochirurgia (SIUEC). L’Endocrinologo 21:359–366. 10.1007/s40619-020-00779-z

[9] Dionigi G, Lombardi D, Lombardi CP, Carcoforo P, Boniardi M, Innaro N, Chiofalo MG, Cavicchi O, Biondi A, Basile F, Zaccaroni A, Mangano A, Leotta A, Lavazza M, Calò PG, Nicolosi A, Castelnuovo P, Nicolai P, Pezzullo L, De Toma G, Bellantone R, Sacco R, Working Group for Neural Monitoring in Thyroid and Parathyroid Surgery in Italy (2014) Intraoperative neuromonitoring in thyroid surgery: a point prevalence survey on utilization, management, and documentation in Italy. Updates Surg 66(4):269–276. 10.1007/s13304-014-0275-y 25465057 10.1007/s13304-014-0275-y

[10] Choi SY, Son YI (2019) Intraoperative neuromonitoring for thyroid surgery: the proven benefits and limitations. Clin Exp Otorhinolaryngol 12(4):335–336. 10.21053/ceo.2019.00542 31575106 10.21053/ceo.2019.00542 PMC6787475

[11] Ling Y, Zhao J, Zhao Y, Li K, Wang Y, Kang H (2020) Role of intraoperative neuromonitoring of recurrent laryngeal nerve in thyroid and parathyroid surgery. J Int Med Res 48(9):300060520952646. 10.1177/0300060520952646 32961083 10.1177/0300060520952646 PMC7513400

[12] Raffaelli M, Voloudakis N, Barczynski M, Brauckhoff K, Durante C, Gomez-Ramirez J, Koutelidakis I, Lorenz K, Makay O, Materazzi G, Pandev R, Randolph GW, Tolley N, Vriens M, Musholt T (2024) European Society of Endocrine Surgeons (ESES) consensus statement on advanced thyroid cancer: definitions and management. Br J Surg 111(8):znae199. 10.1093/bjs/znae199 39158073 10.1093/bjs/znae199 PMC11331340

[13] Ji S, Hu M, Zhang C, Pei M (2024) Systematic review with meta-analysis of intraoperative neuromonitoring during thyroid reoperation. Pak J Med Sci 40(8):1860–1866. 10.12669/pjms.40.8.8241 39281237 10.12669/pjms.40.8.8241 PMC11395382

[14] Randolph GW, Dralle H, International Intraoperative Monitoring Study Group, Abdullah H, Barczynski M, Bellantone R, Brauckhoff M, Carnaille B, Cherenko S, Chiang FY, Dionigi G, Finck C, Hartl D, Kamani D, Lorenz K, Miccolli P, Mihai R, Miyauchi A, Orloff L, Perrier N, Poveda MD, Romanchishen A, Serpell J, Sitges-Serra A, Sloan T, Van Slycke S, Snyder S, Takami H, Volpi E, Woodson G (2011) Electrophysiologic recurrent laryngeal nerve monitoring during thyroid and parathyroid surgery: international standards guideline statement. Laryngoscope 121(Suppl 1):S1-16. 10.1002/lary.21119 21181860 10.1002/lary.21119

[15] Fassari A, Micalizzi A, Lelli G, Gurrado A, Polistena A, Iossa A, De Angelis F, Martini L, Tamagnini GT, Testini M, Cavallaro G (2024) impact of intermittent intraoperative neuromonitoring (IONM) on the learning curve for total thyroidectomy by residents in general surgery. Surg Innov 31(4):355–361. 10.1177/15533506241248974 38632109 10.1177/15533506241248974

[16] Horne SK, Gal TJ, Brennan JA (2007) Prevalence and patterns of intraoperative nerve monitoring for thyroidectomy. Otolaryngol Head Neck Surg 136(6):952–956 17547986 10.1016/j.otohns.2007.02.011

[17] Sturgeon C, Sturgeon T, Angelos P (2009) Neuromonitoring in thyroid surgery: attitudes, usage patterns, and predictors of use among endocrine surgeons. World J Surg 33(3):417–425 18758849 10.1007/s00268-008-9724-4

[18] Ho Y, Carr MM, Goldenberg D (2013) Trends in intraoperative neural monitoring for thyroid and parathyroid surgery amongst otolaryngologists and general surgeons. Eur Arch Otorhinolaryngol 270(9):2525–2530. 10.1007/s00405-013-2359-6 23371538 10.1007/s00405-013-2359-6

[19] Dralle H, Sekulla C, Lorenz K, NguyenThanh P, Schneider R, Machens A (2012) Loss of the nerve monitoring signal during bilateral thyroid surgery. Br J Surg 99(8):1089–1095. 10.1002/bjs.8831 22696115 10.1002/bjs.8831

[20] Hopkins C, Khemani S, Terry RM, Golding-Wood D (2005) How we do it: nerve monitoring in ENT surgery: current UK practice. Clin Otolaryngol 30(2):195–198 15839876 10.1111/j.1365-2273.2004.00933.x

[21] Poveda MD, Martos Martínez JM, Vidal Pérez O, Gluckmann Maldonado E, Quintana De la Basarrate A, Del Moral JV, Rodríguez-Caravaca G (2024) Patterns and indications of intraoperative nerve monitoring usage during thyroidectomy and parathyroidectomy in Spain: results of a national survey of endocrine surgeons. Sci Rep 14(1):17680. 10.1038/s41598-024-68230-z 39085408 10.1038/s41598-024-68230-z PMC11291499

[22] Barczyński M, Randolph GW, Cernea C, International Neural Monitoring Study Group in Thyroid and Parathyroid Surgery (2016) International survey on the identification and neural monitoring of the EBSLN during thyroidectomy. Laryngoscope 126(1):285–291. 10.1002/lary.25548 26452247 10.1002/lary.25548

[23] Saxe A, Idris M, Gemechu J (2024) Does the use of intraoperative neuromonitoring during thyroid and parathyroid surgery reduce the incidence of recurrent laryngeal nerve injuries? A systematic review and meta-analysis. Diagnostics (Basel) 14(9):860. 10.3390/diagnostics14090860 38732275 10.3390/diagnostics14090860 PMC11083343

[24] Patel KN, Yip L, Lubitz CC, Grubbs EG, Miller BS, Shen W, Angelos P, Chen H, Doherty GM, Fahey TJ 3rd, Kebebew E, Livolsi VA, Perrier ND, Sipos JA, Sosa JA, Steward D, Tufano RP, McHenry CR, Carty SE (2020) Executive summary of the American association of endocrine surgeons guidelines for the definitive surgical management of thyroid disease in adults. Ann Surg 271(3):399–410. 10.1097/SLA.0000000000003735 32079828 10.1097/SLA.0000000000003735

[25] Huang TY, Tseng HY, Frattini F, Russell MD, Ahmed AHA, Weber F, Wierzbicka P, Lu IC, Jung KY, Makay Ö, Chai YJ, Chiang FY, Schneider R, Barczyński M, Dralle H, Randolph GW, Wu CW, Dionigi G (2024) The INMSG survey on the loss of signal management on the first side during planned bilateral thyroid surgery. J Otolaryngol Head Neck Surg 53:19160216241265684. 10.1177/19160216241265684 39092609 10.1177/19160216241265684 PMC11378345

[26] Ramesh S, Van Den Berg NH, Sheahan P (2024) Outcomes of immediate total thyroidectomy in first-side loss of neuromonitoring signal. JAMA Otolaryngol Head Neck Surg 150(6):509–516. 10.1001/jamaoto.2024.0698 38662382 10.1001/jamaoto.2024.0698 PMC11046407

[27] Karpathiotakis M, D’Orazi V, Ortensi A, Biancucci A, Melcarne R, Borcea MC, Scorziello C, Tartaglia F (2022) Intraoperative neuromonitoring and optical magnification in the prevention of recurrent laryngeal nerve injuries during total thyroidectomy. Medicina (Kaunas) 58(11):1560. 10.3390/medicina58111560 36363517 10.3390/medicina58111560 PMC9692813

[28] Calò PG, Pisano G, Medas F, Pittau MR, Gordini L, Demontis R, Nicolosi A (2014) Identification alone versus intraoperative neuromonitoring of the recurrent laryngeal nerve during thyroid surgery: experience of 2034 consecutive patients. J Otolaryngol Head Neck Surg 43(1):16. 10.1186/1916-0216-43-16 24942225 10.1186/1916-0216-43-16 PMC4074847

[29] Dralle H, Lorenz K, Machens A (2012) Verdicts on malpractice claims after thyroid surgery: emerging trends and future directions. Head Neck 34(11):1591–1596. 10.1002/hed.21970 22431167 10.1002/hed.21970

[30] Angelos P (2012) Ethical and medicolegal issues in neuromonitoring during thyroid and parathyroid surgery: a review of the recent literature. Curr Opin Oncol 24(1):16–21. 10.1097/CCO.0b013e32834cd596 22051523 10.1097/CCO.0b013e32834cd596

[31] Wu CW, Huang TY, Randolph GW, Barczyński M, Schneider R, Chiang FY, Silver Karcioglu A, Wojtczak B, Frattini F, Gualniera P, Sun H, Weber F, Angelos P, Dralle H, Dionigi G (2021) Informed consent for intraoperative neural monitoring in thyroid and parathyroid surgery - consensus statement of the international neural monitoring study group. Front Endocrinol (Lausanne) 12:795281. 10.3389/fendo.2021.795281 34950109 10.3389/fendo.2021.795281 PMC8689131

[32] Choi S, Shin S, Lee W, Choi SM, Kang SW (2020) Medicolegal lessons learned from thyroidectomy-related lawsuits: an analysis of judicial precedents in South Korea From 1998 to 2019. Gland Surg 9(5):1286–1297. 10.21037/gs-20-398 33224803 10.21037/gs-20-398 PMC7667072

[33] Demontis R, Pittau MR, Maturo A, Petruzzo P, Calò G (2017) Medico legal aspects on neuromonitoring in thyroid surgery: informed consent on malpractice claims. G Chir 38(3):149–154. 10.11138/gchir/2017.38.3.149 29205147 10.11138/gchir/2017.38.3.149 PMC5726504

[34] Verzeletti A, Vassalini M, Bin P, Lancini L, Restori M, De Ferrari F (2016) Malpractice claims related to recurrent laryngeal nerve injury: forensic remarks regarding 15 cases. Egypt J Forensic Sci 6(4):501–504. 10.1016/j.ejfs.2016.04.001

[35] Ferguson BD, Angelos P (2021) Ethical and legal considerations of patients audio recording, videotaping, and broadcasting physician encounters. JAMA Surg 156(2):119–120. 10.1001/jamasurg.2020.2968 33084856 10.1001/jamasurg.2020.2968

[36] Alesina PF, Hinrichs J, Meier B, Cho EY, Bolli M, Walz MK (2014) Intraoperative neuromonitoring for surgical training in thyroid surgery: its routine use allows a safe operation instead of lack of experienced mentoring. World J Surg 38(3):592–598. 10.1007/s00268-013-2372-3 24305928 10.1007/s00268-013-2372-3

[37] Gallo G, Guaitoli E, Barra F, Picciariello A, Pasculli A, Coppola A, Pertile D, Meniconi RL, SPIGC Surgical Training Working Group (2023) Restructuring surgical training after COVID-19 pandemic: a nationwide survey on the Italian scenario on behalf of the Italian polyspecialistic young surgeons society (SPIGC). Front Surg 9:1115653. 10.3389/fsurg.2022.1115653 36713665 10.3389/fsurg.2022.1115653 PMC9875563

[38] Serenari M, Colonnello V, Ratti F, Pertile D, Meniconi RL, Mazzari A, Magnavita N, Russo PM, Italian Polyspecialistic Society of Young Surgeons (SPIGC) (2023) The state of general surgery residents in Italy after COVID-19 outbreak: a nationwide cross-sectional study. Updates Surg 75(1):95–103. 10.1007/s13304-022-01370-x 36057026 10.1007/s13304-022-01370-x PMC9440313

[39] Karelitz TM, Budescu DV (2004) You say “probable” and I say “likely”: improving interpersonal communication with verbal probability phrases. J Exp Psychol Appl 10(1):25–41. 10.1037/1076-898X.10.1.25 15053700 10.1037/1076-898X.10.1.25

[40] Sur MD, Angelos P (2016) Ethical issues in surgical critical care: the complexity of interpersonal relationships in the surgical intensive care unit. J Intensive Care Med 31(7):442–450. 10.1177/0885066615585953 25990272 10.1177/0885066615585953

[41] Hashim MJ (2024) Verbal probability terms for communicating clinical risk - a systematic review. Ulster Med J 93(1):18–23 38707974 PMC11067312

[42] Bomhof-Roordink H, Gärtner FR, Stiggelbout AM, Pieterse AH (2019) Key components of shared decision-making models: a systematic review. BMJ Open. 10.1136/bmjopen-2019-031763 31852700 10.1136/bmjopen-2019-031763 PMC6937101

