# Supplementary material for: Intraoperative nerve monitoring in thyroid and parathyroid surgery: a decade of Italian practice
Source: Updates Surg. 2025 Apr 1;77(5):1563–79. doi: 10.1007/s13304-025-02157-6 (PMC12420727; doi:10.1007/s13304-025-02157-6)
Supplement: Supplementary file 1 — Supplementary file1 (PDF 326 KB) [file 13304_2025_2157_MOESM1_ESM.pdf]

QUESTIONARIO: Utilizzo IONM del NLR in Italia *oggi* -**Update Survey 2014**-

Utilizzo Intraoperative Neuromonitoring del Nervo Laringeo Ricorrente in Italia oggi.

* Indica una domanda obbligatoria

1. Email *

____

Informazioni generali

2. Attualmente il Centro in cui pratica la Sua attività chirurgica su trova nel: *

Contrassegna solo un ovale.

- ( ) Nord Italia
- ( ) Centro Italia
- ( ) Sud Italia o Isole
- ( ) Altro: ________________________________

3. Il Centro presso il quale lavora attualmente afferisce ad una Struttura: *

Contrassegna solo un ovale.

- ( ) Universitaria
- ( ) Ospedaliera
- ( ) Privata convenzionata
- ( ) altro

4. Il Centro di chirurgia endocrina presso il quale svolge la Sua attività si può definire una: *

Contrassegna solo un ovale.

- Unità Operativa Complessa (UOC)
- Unità Operativa Semplice (UOS)
- Programma di Chirurgia
- Altro

5. Da quanti anni circa è stato istituito il Centro di chirurgia endocrina presso la struttura in cui presta servizio? *

Indichi cortesemente il *numero* di anni circa.

____

6. Qual è il numero di chirurghi strutturati che afferisce al Suo Centro e che si occupa di chirurgia endocrina? *

Indichi cortesemente il *numero* di chirurghi strutturati.

____

7. Qual è l'età media dei chirurghi che opera presso il Centro di chirurgia presso cui presta servizio?

Indichi cortesemente l'*età media* circa.

____

8. Nella Sua equipe operatoria c'è una preferenza per l'utilizzo dello strumento (che si tramuta in effettivo maggiore utilizzo) da parte di uno o più operatori rispetto ad altri?

Contrassegna solo un ovale.

- Sì
- No
- Non mi sembra

9. Nel caso in cui abbia risposto “Sì” alla domanda precedente, ha notato se questa predisposizione all’utilizzo della tecnologia sia legata ad un fattore di età? (*es. età più giovane, maggiore predisposizione all’utilizzo di tecnologie*)

Nel caso in cui voglia segnalare la possibile associazione con altri fattori, riporti lo stesso in "altro"

Contrassegna solo un ovale.

- Sì
- No
- Non mi sembra
- Altro

Attività di chirurgia endocrina

10. Quanti interventi di tiroidectomia totale vengono effettuati nel Suo Centro ***all’anno*** approssimativamente? *

Contrassegna solo un ovale.

- ≤50
- >50-150
- >150-250
- >250-350
- >350-500
- >500

11. Quanti interventi di paratiroidectomia vengono effettuati nel Suo Centro ***all'anno*** approssimativamente? *

Contrassegna solo un ovale.

- ≤50
- >50-150
- >150-250
- >250-350
- >350-500
- >500

12. Quanti interventi di svuotamenti linfonodali del collo vengono effettuati nel Suo Centro ***all'anno*** approssimativamente? *

Contrassegna solo un ovale.

- ≤50
- >50-150
- >150-250
- >250-350
- >350-500
- >500

13. Quanti interventi di chirurgia endocrina (in generale) effettua ***in un mese*** con la Sua Equipe? Indichi cortesemente un numero *

____

14. Se pensa alla Sua attività di chirurgia endocrina (e quella della Sua Equipe) degli ultimi 10 anni, ritiene che l'utilizzo dell'IONM del NLR abbia *oggi* più ampio impiego nella pratica chirurgica, rispetto al 2014? *

Contrassegna solo un ovale.

- Sì
- No
- più o meno uguale

15. Quali pensa che siano i motivi che hanno reso l'utilizzo dell'IONM del NLR ad oggi più diffuso nella comune pratica di chirurgia endocrina? Cortesemente indichi quelle che sono, a Suo avviso, le principali motivazioni. *

Indichi cortesemente *almeno tre* motivazioni

Gestione pre-operatoria al paziente candidato a chirurgia endocrina

16. Quale delle seguenti metodiche di valutazione preoperatoria descrive meglio il Suo approccio all'*esame della laringe* prima della chirurgia tiroidea?

Contrassegna solo un ovale.

- Esecuzione routinaria di fibrolaringoscopia diretta
- Esecuzione in casi selezionati di fibrolaringoscopia diretta
- Esecuzione routinaria di fibrolaringoscopia indiretta e, in casi selezionati, di fibrolaringoscopia diretta
- Non viene mai eseguito un esame pre-operatorio di laringoscopia
- Altro:

17. Qualora abbia indicato "*altro*" come risposta alla domanda precedente, potrebbe cortesemente riportare, in maniera sintetica, la risposta che avrebbe ritenuto opportuna?

18. Nel Suo Centro, il **consenso informato** all'atto chirurgico viene somministrato al paziente da: *

Contrassegna solo un ovale.

- medico specializzando in formazione da solo
- medico specializzando in formazione tutorato da un medico strutturato
- medico strutturato
- Referente dell'Unità di chirurgia endocrina
- Altro

19. Nel Suo Centro, viene utilizzato il consenso informato reso disponibile dalla Società Italiana di chirurgia (SIC)? https://www.chirurgia-endocrina.it/app/download/16027632/ConsensoTiroide.pdf *

Contrassegna solo un ovale.

- Sì
- No
- Sì, ma sono state apportate alcune modifiche

20. Nel caso in cui avesse sentito la necessità di apportare delle modifiche, cortesemente potrebbe indicarci sinteticamente quali?

21. Durante la somministrazione del consenso informato all'atto chirurgico, qual è il Suo approccio alla **comunicazione del rischio** di danno al nervo laringeo ricorrente? Cortesemente può descrivere brevemente quelle che sono le parole chiave che utilizza per nella Sua comunicazione. *

Elencare cortesemente *almeno tre* parole chiave, in *ordine* di *importanza*.

22. Crede che poter riferire al paziente che durante l'intervento chirurgico verrà utilizzato il monitoraggio intraoperatorio del nervo laringeo, Le sia di ausilio per rendere *più efficace* la Sua **comunicazione**? *

Contrassegna solo un ovale.

- Sì
- No
- Indifferente
- Altro: ____________

23. Crede che poter riferire al paziente che durante l'intervento chirurgico verrà utilizzato il monitoraggio intraoperatorio del nervo laringeo, sia *rassicurante* per il **chirurgo**?

Contrassegna solo un ovale.

- Sì
- No
- Indifferente
- Altro: ____________

24. Crede che poter riferire al paziente che durante l'intervento chirurgico verrà utilizzato il monitoraggio intraoperatorio del nervo laringeo, *tranquillizzi* maggiormente il **paziente**?

Contrassegna solo un ovale.

- Sì
- No
- Indifferente
- Altro: ______

Vantaggi e svantaggi IONM del NLR

25. Le capita che il ***paziente domandi*** se lo strumento del neuromonitoraggio intraoperatorio *verrà utilizzato* per il tipo di chirurgia a cui verrà sottoposto?

Contrassegna solo un ovale.

- Sempre
- Sì, spesso
- Sì, a volte
- Raramente
- No, mai

26. Quale ritiene sia l'**utilità principale** del monitoraggio del nervo laringeo? Selezioni *tutte le opzioni* che ritiene pertinenti. *

Seleziona tutte le voci applicabili.

- [ ] Ritengo che sia utile per la gestione del nervo
- [ ] Credo che sia utile per ridurre le lesioni permanenti del NLR
- [ ] Credo che sia utile per ridurre le lesioni temporanee del NLR
- [ ] Rassicurazione del paziente
- [ ] Rassicurazione del chirurgo
- [ ] Finalità di formazione
- [ ] Penso che non abbia alcuna utilità
- [ ] Tutte le precedenti
- [ ] Altro: ______________________

27. Ritiene che tra le opzioni elencate nella domanda precedente ***non** fosse riportata* una indicazione a Suo avviso rilevante? In caso affermativo, *riporti con una breve risposta*, quale indicazione ritiene opportuno aggiungere alle precedenti.

____

____

____

____

____

28. Utilizza il neuromonitoraggio del NLR durante ***tutti*** gli interventi di chirurgia endocrina sul collo?

Contrassegna solo un ovale.

- ( ) Sì, in tutte le operazioni
- ( ) No, non lo utilizzo sempre, ma lo utilizzo spesso
- ( ) No, lo utilizzo in casi selettivi
- ( ) No, l'ho usato diverse volte ma abbandonato
- ( ) No, non ho mai provato

29. In ***quali situazioni*** utilizza l'IONM del NLR? Selezionare *tutte* quelle che si applicano.

Seleziona tutte le voci applicabili.

- [ ] Gozzo retrosternale
- [ ] Carcinoma della tiroide
- [ ] Individuazione preoperatoria della paralisi ccvv
- [ ] Reinterventi, casi complessi
- [ ] Il paziente ha una voce normale ma riferisce "raucedine"
- [ ] Il paziente richiede il neuromonitoraggio
- [ ] Svuotamento centrale e laterocervicale del collo
- [ ] Malattia di Graves
- [ ] Disfonia preoperatoria
- [ ] Chirurgia robotica
- [ ] Sempre
- [ ] Altro: ______________________

30. Qualora il Suo sia un utilizzo "**selettivo**", dunque non si avvale dell'ausilio dello strumento ad ogni intervento chirurgico, quali ritiene siano i casi in cui questo risulta *essenziale*?

Descriva *massimo tre casi* in cui ritiene che l'ausilio dell'IONM del NLR sia indispensabile

____

____

____

____

____

31. Utilizza il neuromonitoraggio durante la tiroidectomia per l'**identificazione** del NLR?

Contrassegna solo un ovale.

- ( ) Sì
- ( ) No

32. Utilizza il neuromonitoraggio durante la tiroidectomia come "**conferma**" del NLR (localizzazione, integrità, decorso, etc.)?

Contrassegna solo un ovale.

- Sì
- No

33. Utilizza il neuromonitoraggio in modalità "**continua**" o "**intermittente**"?

Contrassegna solo un ovale.

- solo continua
- solo intermittente
- dipende

34. Qualora abbia risposto "*dipende*", potrebbe spiegare brevemente in quali situazioni preferisce utilizzare una modalità rispetto all'altra?

Descriva in maniera breve quando utilizza il neuromonitoraggio continuo vs intermittente, motivandolo.

35. Riflettendo in merito alla Sua esperienza personale e della Sua Equipe con l'utilizzo dell'IONM del NRL in chirurgia tiroidea e paratiroidea, ricorda di aver avuto casi di ***falsi positivi*** (segnale i.o. di nervo "leso" e assenza di parali cordale p.o.) e/o ***falsi negativi*** (segnale di nervo "sano" i.o. e paralisi cordale p.o.)?

Contrassegna solo un ovale.

- Sì
- No
- Non ricordo

36. Qualora abbia fornito risposta affermativa alla domanda precedente, potrebbe indicare percentualmente quante volte questo è capitato nel corso dello scorso anno?

Pensando all'attività chirurgica del *2023* del Suo Centro, indichi cortesemente *in quanti casi su 100* questo evento si è verificato.

____

37. Qualora durante un intervento chirurgico di **tiroidectomia totale**, lo strumento di neuromonitaoraggio intraoperatorio le segnalasse una "*lesione del nervo*" ricorrente durante lo step chirurgico di lobectomia sul *primo lobo*, quale sarebbe il Suo atteggiamento?

Indichi quale delle seguenti affermazioni descrive meglio il Suo pensiero. (es. decido sempre di fermarmi?, ha più "peso" la valutazione visiva del nervo? o la valutazione della clinica di partenza?)

Seleziona tutte le voci applicabili.

- [ ] Mi fermo e programmo una "two-stage thyroidectomy"
- [ ] Valuto con attenzione "visivamente" il nervo e decido con la mia Equipe se proseguire o meno (es. se visivamente il nervo appare "integro", scelgo di continuare)
- [ ] In considerazione del quadro clinico del paziente, decido con la mia Equipe se proseguire o meno, anche in caso di evidente danno anatomico e perdita di segnale
- [ ] Altro

38. Ritiene che tra le opzioni elencate nella domanda precedente ***non*** *fosse riportata* una affermazione a Suo avviso rilevante? In caso affermativo, *riporti con una breve risposta*, quale valutazione ritiene opportuno aggiungere alle precedenti.

Questi contenuti non sono creati né avallati da Google.

Google Moduli
