# Supplementary material for: Intraoperative nerve monitoring in thyroid and parathyroid surgery: a decade of Italian practice
Source: Updates Surg. 2025 Apr 1;77(5):1563–79. doi: 10.1007/s13304-025-02157-6 (PMC12420727; doi:10.1007/s13304-025-02157-6)
Supplement: Supplementary file 2 — Supplementary file2 (DOCX 10 KB) [file 13304_2025_2157_MOESM2_ESM.docx]

**List of Centers Participating in the Survey on the Use of IONM – SIUEC 2024**

1. ASST Grande Ospedale Metropolitano Niguarda, Milano
2. ASST Santi Paolo e Carlo, Milano
3. IRCSS Ospedale San Raﬀaele Milano – Gruppo san Donato, Milano
4. ASST Monza, Ospedale San Gerardo, Milano
5. ASST Fatebenefratelli Sacco, Milano
6. Osp. Vizzolo Predabissi - ASST Melegnano e della Martesana, Milano
7. Casa di Cura Igea, Milano
8. Clinica San Martino, Malgrate (Lecco)
9. Humanitas Mater Domini, Varese
10. Istituto Madre Rubatto, Bergamo
11. DSCS-c/o U.O. Chirurgia Generale 1 - Spedali Civili – Università degli Studi di Brescia, Brescia
12. ASST Ovest Milanese, Legnano, Milano
13. Ospedale Maria Vittoria, Torino
14. Clinica Fornaca di Sessant, Torino
15. Ospedale Humanitas Gradenigo Piemonte, Torino
16. A. O. Ordine Mauriziano di Torino, Torino
17. Istituti Clinici Scientiﬁci Maugeri IRCCS, Pavia
18. Az. Sanitaria Locale Ciriè, Chivasso e Ivrea, Ivrea
19. ASL VCO Ospedale "San Biagio", Domodossola (Verbania)
20. Ospedale S. Lazzaro, Alba (Cuneo)
21. Azienda Sanitaria dell’Alto Adige, Brunico
22. Ospedale San Martino, Genova
23. Az. Ospedaliera Universitaria di Padova, Padova
24. Casa di Cura polispecialistica Dott. Pederzoli, Verona
25. Azienda Ospedaliera Universitaria Integrata Verona, Verona
26. Az. Sanitaria Universitaria Giuliano Isontina, Trieste
27. Azienda Ospedaliero-Universitaria di Ferrara, Ferrara
28. Az. Ospedaliera Universitaria di Parma, Parma
29. Ospedale Civile Baggiovara, Modena
30. AUSL della Romagna, Forlì
31. Az. Ospedaliero-Universitaria Pisana, Pisa
32. Ospedale «Felice Lotti», Pontedera, Pisa
33. Ospedale SS Cosma e Damiano, Pescia, Prato
34. Az. Ospedaliero-Universitaria Careggi, Firenze
35. Ospedale Santa Maria Nuova – Az. Usl Toscana Centro, Firenze
36. Casa di Cura Villa Donatello, Firenze
37. Az. Ospedaliera Santa Maria Terni, Terni
38. Ospedale Carlo Urbani di Jesi, Jesi (Ancona)
39. Casa di Cura Pierangeli, Pescara
40. Ospedale Santo Spirito di Pescara, Pescara
41. Fondazione policlinico Universitario Agostino Gemelli, Roma
42. Ospedale Fatebenefratelli-Isola Tiberina, Roma
43. AOU Policlinico Umberto I –Dipartimento di Chirurgia Generale e Specialistica, Roma
44. AOU Policlinico Umberto I –Dipartimento di Chirurgia Generale, Roma
45. AOU Sant’Andrea di Roma, Roma
46. Ospedale San Carlo di Nancy, Roma
47. Ospedale Cristo Re, Roma
48. IDI IRCCS, Roma
49. Policlinico Casilino, Roma
50. Ospedale Sant'Eugenio ASL Roma 2, Roma
51. Ambulatorio Ospedaliero di Endocrinochirurgia ASL ROMA 4, Roma
52. Ospedale ICOT Marco Pasquali, Latina
53. Az. Ospedaliera San Pio, Benevento
54. Az. Ospedaliera Universitaria «Luigi Vanvitelli», Napoli
55. AORN Antonio Cardarelli, Napoli
56. IRCCS Fondazione G. Pascale, Napoli
57. ASL NA1 Centro - Ospedale San Paolo, Napoli
58. A.O.R.N. "San Giuseppe Moscati", Avellino
59. AOU San Giovanni di Dio e Ruggi d’Aragona di Salerno, Salerno
60. Az. Ospedaliera Universitaria Policlinico «Giovanni XXIII» di Bari -Unità di Chirurgia Generale Laparoscopica e d'Urgenza, Bari
61. Az. Ospedaliera Universitaria Policlinico «Giovanni XXIII» di Bari -Unità di Chirurgia Generale, Bari
62. U.O.C. di Chirurgia Generale P.O. San Paolo, Bari
63. Az. Ospedaliero Universitaria Mater Domini, Catanzaro
64. Az. Ospedaliera Universitaria di Sassari, Sassari
65. Az. Ospedaliero-Universitaria di Cagliari, Cagliari
66. Humanitas Istituto Clinico Catanese S.p.A., Catania
67. Dipartimento di Chirurgia generale e specialità medico chirurgiche – Università di Catania, Catania
68. Az. Universitaria Policlinico Paolo Giaccone –Ch. Generale ad indirizzo oncologico, Palermo
69. Az. Universitaria Policlinico Paolo Giaccone –Ch. Generale d’Urgenza e Trapianti d’organo, Palermo
70. Fondazione Istituto G. Giglio di Cefalù, Cefalù (Palermo)
